# Percutaneous Embolization for Painful Varicocele: An 8-Year Tertiary Centre Experience

**DOI:** 10.5334/jbsr.3769

**Published:** 2025-02-04

**Authors:** Philippe Haroun, Salvatore Murgo, Georges Mjaess, Thierry Roumeguère, Fadi Tannouri

**Affiliations:** 1 Department of Interventional Radiology, Erasme Hospital, Hôpital Universitaire de Bruxelles, Brussels, Belgium; 2 Urology Department, Erasme Hospital, Hôpital Universitaire de Bruxelles, Université Libre de Bruxelles, Brussels, Belgium

**Keywords:** Varicocele, percutaneous embolization, scrotal pain, interventional radiology

## Abstract

*Background:* Varicocele is a common condition in men that can lead to several clinical problems. Treatment modalities include surgical and non‑surgical methods. There has been no randomized controlled trial proving the superiority of one treatment approach over another.

*Materials and methods:* We conducted an 8‑year retrospective analysis of varicocele embolization procedures at our department of Interventional Radiology. Demographic data, procedure details, procedure success and complications were collected. Telephone interviews were also conducted at the time of the study.

*Results:* A total of 182 interventions were performed. Median age of patients at presentation was 31 years (range, 12–71). Median follow‑up duration was 44.5 months (range, 3.4–106.9). Employed embolic agents were coils in 171/182 (91.94%) cases. Technical success rate was 88.15%. Ultrasonographic success was accomplished in 44.87% patients. Clinical success rate in patients referred for scrotal pain was 70.42%. Fluoroscopy time was 20.3 ± 14.9 min (mean ± SD), dose area product was 63.2 ± 50.5 Gy∙cm² (mean ± SD) and kinetic energy released per unit mass was 193.2 ± 173.6 (mean ± SD). Minor complications were encountered in 19/182 (10.45%) of the interventions.

*Conclusions:* Varicocele embolization was found to be an efficient and safe procedure for patients referred for scrotal pain. Randomized controlled trials are warranted to elaborate treatment algorithms in varicocele patients.

## I. Introduction

Varicocele is an abnormal enlargement and tortuosity of the pampiniform plexus associated with retrograde blood flow through the internal spermatic vein (ISV) [1, 2]. Varicocele affects around 15% of adolescents and adults [1, 3]. Varicocele is understood to have a multifactorial aetiology, with the most widely accepted explanation being congenital or acquired valve dysfunction [1]. Other recognized mechanisms include anatomical variations of the ISV and left venous obstruction by the ‘nutcracker phenomenon’ [1, 4, 5].

The majority of men with varicocele have no symptoms and are identified on routine or self‑examination [1, 6]. Clinical presentations include painful varicocele, infertility and asymmetric testicular development [1, 6–10]. An estimated 2–10% present with scrotal pain and have significant repercussions on quality of life [1, 2, 7, 8]. Varicocele is widely considered to be the most common correctable cause of male infertility [6–8].

At presentation, the laterality distribution for left‑sided varicocele, right‑sided varicocele and bilateral varicocele are 80–90%, 5–10% and 1–15%, respectively [8]. The majority of varicoceles are left‑sided, primarily owing to the venous drainage of the ISV to the left renal vein at a sharp angle [1].

To grade varicocele, both clinical and ultrasound findings may be employed. Amongst the most widely used classifications is the Dubin and Amelar clinical grading system [11]. For ultrasonographic examination, the grading system proposed by Sarteschi is the most widely accepted [1, 12].

The ultimate goal of varicocele repair is the interruption of reflux through the ISV [2]. This can be achieved by surgical as well as non‑surgical means (percutaneous varicocele embolization or sclerotherapy). To date, there has been no randomized controlled trial proving the superiority of one treatment modality over another [6–11].

Percutaneous varicocele embolization is the least invasive approach, as it is performed in an outpatient setting under local anaesthesia [1–13]. It eliminates the risks associated with general anaesthesia and technically removes the risk of testicular artery damage [13]. Besides, this approach offers the ability to perform intra‑operative venography, enabling the identification of ISV variants and collateral venous vascular supply contributing to clinical pathology [13]. However, it demands interventional radiologic expertise and has potentially serious, yet rare, complications [6, 13]. It also involves radiation exposure and the possibility of contrast media reaction [1].

The primary endpoints of our study were the technical success, ultrasonographic success, clinical success in the setting of patients referred for scrotal pain and complication rates of percutaneous varicocele embolization. The secondary endpoint included a dosimetric study.

## II. Materials and Methods

This is a retrospective study of a consecutive series of patients referred to our interventional radiology department for percutaneous varicocele embolization from 2014 to 2022. After the Ethics Committee’s approval and following the principles of the Declaration of Helsinki, institutional clinical records and procedure reports were explored to collect the following information: demographic data, indication of the procedure, venous access point, technical success, dosimetric parameters (fluoroscopy duration, dose area product and kinetic energy released per unit mass) and ultrasonographic recurrence at 3–6 months.

With informed verbal consent, telephone interviews were conducted at the time of the study to get an update on the patients’ clinical situation. Patients referred for scrotal pain were required to evaluate scrotal pain prior to and then following the intervention on a visual analogue scale from 0 (no pain) to 10 (worst possible pain). In the case of partial pain improvement, patients were asked to clarify whether they were satisfied with the procedure or not.

Clinical success was evaluated only in patients referred for scrotal pain. In this category of patients, clinical success was considered to be accomplished in patients with complete pain resolution and in patients with partial pain relief accompanied by satisfaction.

Ultrasonographic success was defined as a complete cessation of spermatic venous reflux (SVR) on Doppler ultrasound at 3–6 months.

### Embolization procedure

Procedures were conducted in an outpatient setting by experienced interventional radiologists or under their supervision.

**Figure 1:**
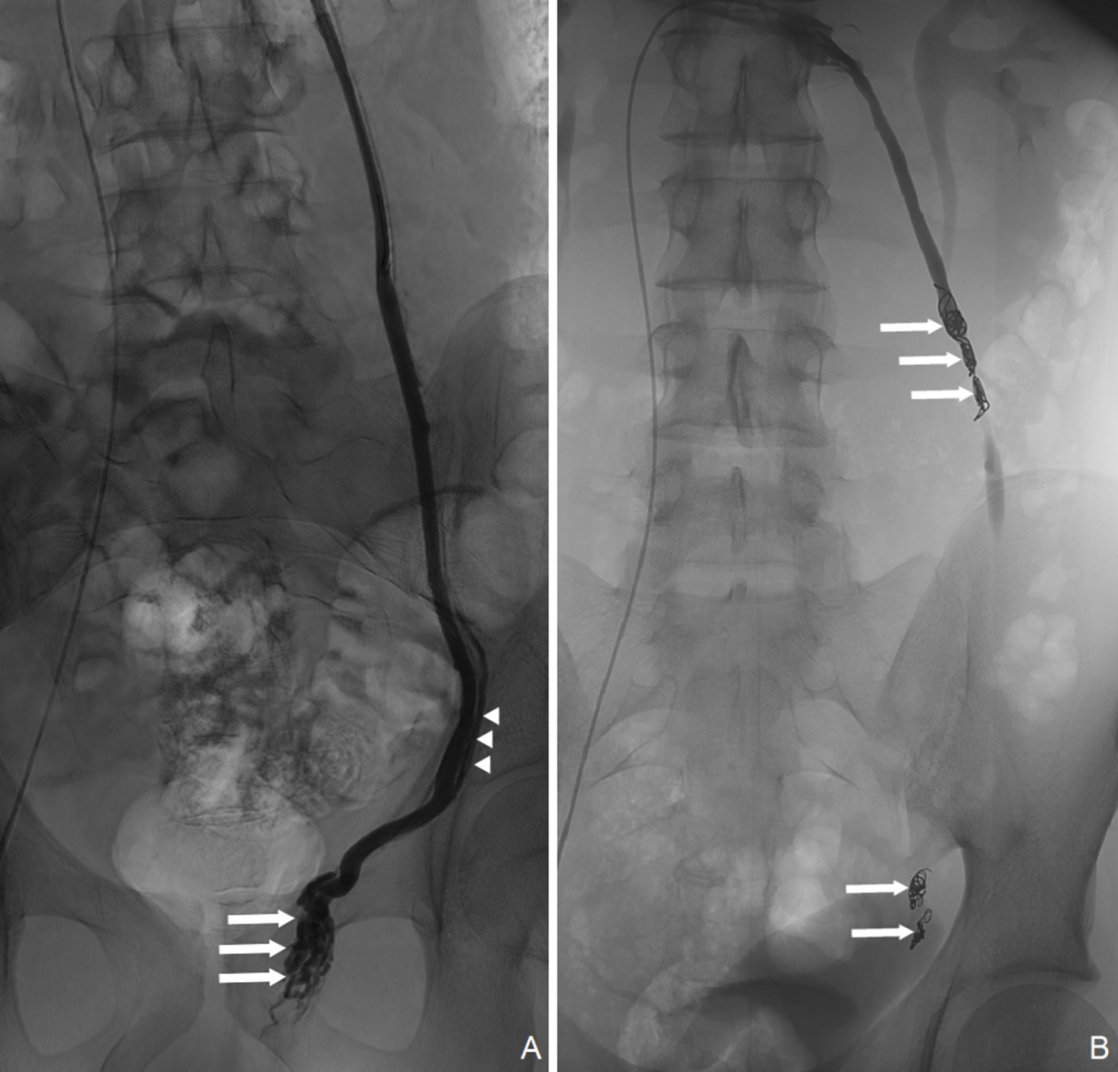
Fluoroscopy image showing selective catheterization of an insufficient left ISV during Valsalva manoeuvre before embolization **(A)**, noting dilated veins in the pampiniform plexus (arrows) and the presence of a collateral vein (arrowheads). Fluoroscopy image showing a sandwich coil embolization at two levels of the left ISV (arrows) resulting in a complete stasis of contrast media at the proximal level **(B)**.

After local anaesthesia, venous access was obtained at the right common femoral level or more rarely at jugular or brachial levels under ultrasound guidance. A 4‑ or 6‑French sheath was placed, and a hydrophilic guidewire was preloaded into a 4‑French catheter. The guidewire advanced under fluoroscopic guidance through the venous system to reach the ISV. At the ostial level of the ISV, injections of contrast media were performed to obtain a mapping of the venous drainage system and to identify incompetent valves (Figure 1A).

Choice of embolic agent was based on operator preference when confronted with venous anatomical variations. For coil embolization, coils were first deployed through the catheter as distally as possible. Then, a sandwich occlusion (Figure 1B) was performed with additional coils in the proximal part of the spermatic vein. Coils deployment and positioning depended on operator choice and venous anatomical variants. Technical success was defined as a cessation of reflux, resulting in complete stasis of contrast media [1, 14]. Patients were observed for 2–4 h before discharge and were advised to avoid strenuous physical activity for 1 week. As part of follow‑up, ultrasound studies were required after 3–6 months.

### Statistical analysis

Statistical analysis was performed using R language for statistical computing [15]. To test normality, the Shapiro–Wilk test was used. The Box–Cox method was utilized to transform data as needed. The threshold for statistical significance was set to *p* < 0.05.

## III. Results

A total of 182 procedures were performed. The median age of patients at presentation was 31 years (range, 12–71). The median follow‑up duration was 44.5 months (range, 3.4–106.9). For a total of 182 patients, the clinical indication for the procedure was scrotal pain in 116 cases (63.73%), infertility in 35 (19.23%) cases, testicular hypotrophy in 6 (3.30%) cases and palpable varicocele in 5 (2.75%) cases, and it was not found in 20 (10.98%) cases.

At presentation, varicocele was left‑sided in 131 cases (71.98%), right‑sided in 2 cases (1.10%) and bilateral in 49 cases (26.92%). In all, 20 ISVs (1 left‑sided, 19 right‑sided) showed normal phlebograms. Hence, a total of 211 insufficient ISVs were subjected to embolization. Technical success was reached in 186 (88.15%) ISVs. The employed embolic agents were coils in 171 cases (91.94%), glue in 3 cases (1.61%) and a combination of coils and glue in 12 cases (6.45%). In single‑sided coil varicocele embolization procedures, 7.6 ± 4.4 (mean ± SD) coils were utilized. The anatomical distribution of insufficient ISVs and technical success rates are presented in Table 1.

**Table 1:** Laterality of varicocele and relative technical success rates.

	LEFT‑SIDED	RIGHT‑SIDED	TOTAL
Number (*n*), (percentage [%])	179 (77.92%)	32 (22.08%)	211 (100%)
Technical success, *n* (%)	167 (93.27%)	19 (59.37%)	186 (88.15%)

### Clinical success

**Figure 2:**
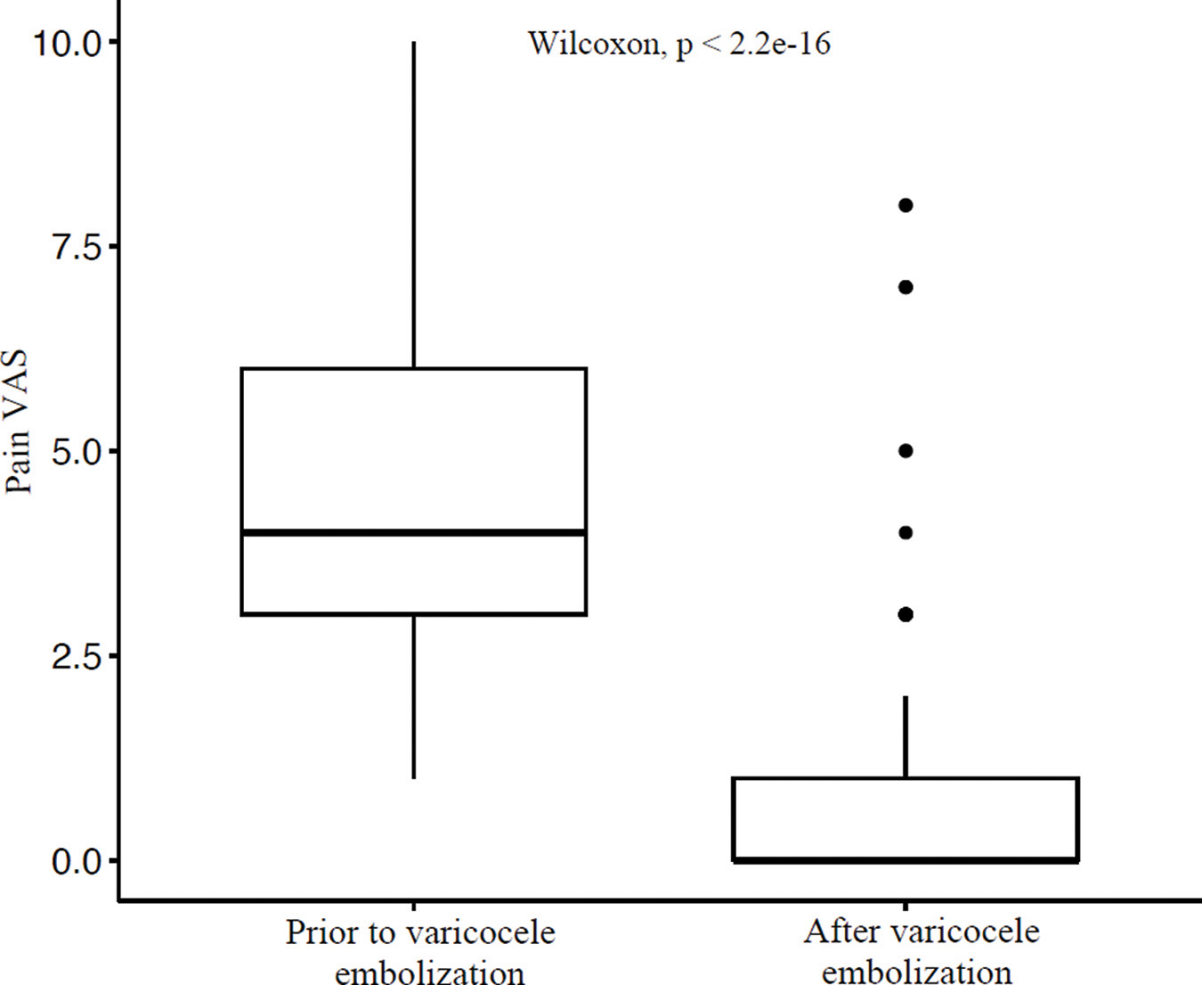
Box and whisker plot for pain VAS before and after the intervention. VAS = Visual Analog Scale.

In patients referred for scrotal pain, 71/116 (61.2%) patients responded to telephone interviews. Pre‑ and post‑embolization pain scores are presented in Figure 2.

**Figure 3:**
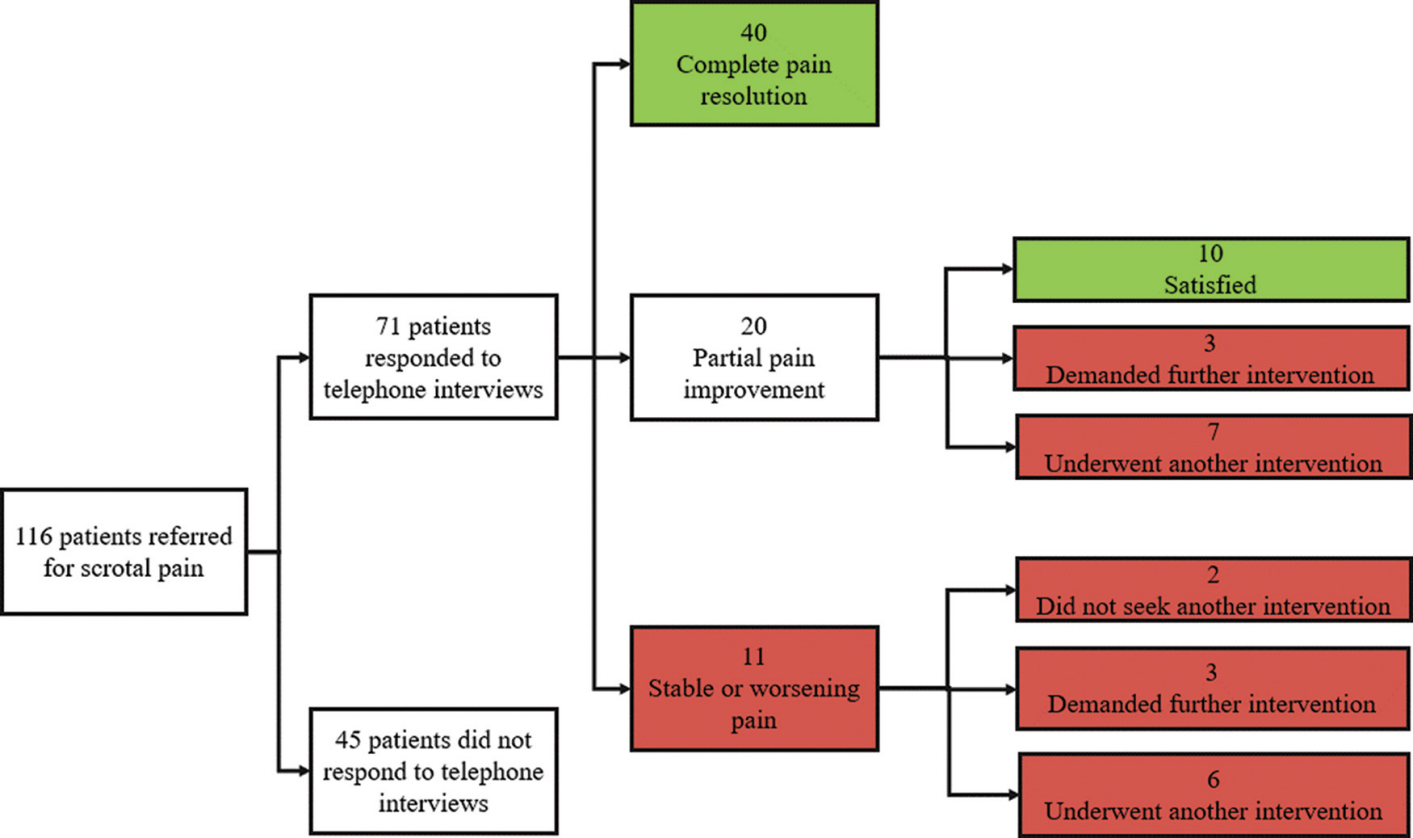
Flow chart of patients who underwent varicocele embolization for scrotal pain. Green = clinical success. Red = clinical failure.

A total of 40, 20 and 11 patients reported complete resolution of scrotal pain, partial relief of pain and stable/worsening pain, respectively. In the group with partial pain relief, 10 were satisfied with their clinical condition, 3 demanded further intervention and 7 had already undergone a supplementary procedure for varicocele. Clinical success for scrotal pain was 70.42% (50/71 patients). A schematic illustration is presented in Figure 3.

In other groups of patients (e.g. patients presenting for infertility or for testicular hypotrophy), clinical success was not evaluated. Patients from other groups were mainly included in the study to have a broader cohort for assessing remaining parameters.

### Ultrasonographic success

Ultrasound follow‑up studies (at 3–6 months) were found in 78/182 patients. Ultrasonographic success was achieved in 35/78 (44.87%) of cases. In the group that was referred for scrotal pain and in whom ultrasonographic recurrence was documented, only 17.24% (5/29 patients) had pain recurrence.

### Complications

Complications were encountered in 19/182 (10.45%) interventions. The most common complication – recorded in six (3.30%) patients – was ISV rupture and contrast extravasation. Access point hematoma, hydrocele formation and acute testicular pain were each recorded in three (1.65%) cases. Vagal attacks were noted in two (1.10%) cases. Right femoral artery puncture was noted in one (0.55%) case and light scrotal pain in the months following embolization in one (0.55%) case. All of these complications were self‑limited and did not necessitate any further intervention. There were no long‑term complications.

### Dosimetric parameters

Dosimetric parameters were found in 83/182 (45.60%) interventions. In 74 cases, coils were employed. In two cases, the embolic agent was glue, and in seven cases, a combination of coils and glue was used. Out of the 74 cases with coil embolization, 71 were performed by right femoral access. For statistical analysis, the 71 interventions performed with coils and by femoral access were considered. In 5/71 cases, embolization was performed bilaterally. Duration of fluoroscopy, dose area product DAP and kinetic energy released per unit mass (KERMA) values are presented in Table 2.

**Table 2:** Mean values of duration of fluoroscopy, DAP and KERMA for the 71 interventions performed with coils and by right femoral access point.

	DURATION OF FLUOROSCOPY (MIN)	DAP (GY∙CM²)	KERMA (MGY)
Median [IQR]	17.1 [10, 24.3]	43.1 [30.8, 94.1]	130.4 [88.2, 254]
Mean ± SD	20.3 ± 14.9	63.2 ± 50.5	193.2 ± 173.6
Mean (95% CI)	20.3 [17.1, 23.6]	63.2 [52.1, 74.2]	193.2 [155.3, 231.2]

DAP = dose area product; KERMA = kinetic energy released per unit mass; IQR = interquartile range; SD = standard deviation; CI = confidence interval.

However, the three procedures using glue had a fluoroscopy time of 12.9 ± 1.7 min, a DAP of 42.2 ± 11.6 Gy∙cm² and a KERMA of 142.1 ± 29.5 mGy (mean ± SD).

## IV. Discussion

In the present study, we evaluated – with a median follow‑up of 44.5 months – varicocele embolization with regard to technical success, clinical success for painful varicocele, ultrasonographic success, radiation exposure and complication rates. Percutaneous varicocele embolization seemed to be an efficient and safe procedure for patients presenting with scrotal pain.

The most employed embolic agents for varicocele embolization in interventional radiology literature are coils, largely due to their availability, easy operability and safety [8, 10]. Most of the interventions in our study were carried out using coils (91.94%).

Our global technical success rate (88.15%) was similar to the ones reported in the interventional radiology literature. Technical success rates are influenced by different factors, the most important one being varicocele laterality. In fact, technical success rates for left‑sided varicocele are usually higher than 90% (93.27% in this study), whereas those for right‑sided varicocele range from 51% to 81.4% (59.37% in this study) [1, 16]. In fact, authors suggest a jugular approach for solitary right‑sided varicocele embolization procedures [16, 17]. Technical success rates from different studies are presented in Table 3.

**Table 3:** Table summarizing different studies with employed embolic agents, venous access points, laterality of varicocele in patients and technical success rates.

STUDY	EMBOLIC AGENT (*N*)	VENOUS ACCESS POINT	VARICOCELE LATERALITY (*N*)	TECHNICAL SUCCESS RATE
Prasivoravong et al. [14] (2014)	Coils	Femoral	Left (47)	100%
Cantoro et al. [18] (2015)	Coils	Femoral	Left (218)	89%
Nabi et al. [19] (2003)	Coils	Femoral	Left (50) Right (15) Bilateral (6)	96%
Shlansky‑Goldberg et al. [20] (1997)	Coils	Femoral or jugular	Left (95) Right (15) Bilateral (63)	88%
Bilreiro et al. [21] (2017)	Coils (103) Glue (26)	Femoral	Left (129)	99% (coils) 100% (glue)

Clinical success is defined as the resolution of clinical problems that led to treatment [1]. Varicocele embolization efficacy or clinical success concerning scrotal pain is reported in different manners. Table 4 summarizes studies evaluating this in patients treated for scrotal pain. In fact, clinical success rates are inconsistent due to the lack of a strict definition for clinical success. We considered clinical success to be accomplished in patients who achieved complete pain resolution and in patients who had partial improvement yet who were satisfied with the procedure. Accordingly, we obtained a 70.42% clinical success rate. Puche‑Sanz and colleagues used a similar definition and reported an 86.9% clinical success rate.

**Table 4:** Table reviewing different studies of varicocele embolization, reporting clinical success or recurrence rates in patients referred for scrotal pain, with follow‑up durations and definitions of the studied entities.

STUDY	FOLLOW‑UP DURATION (MONTHS)	STUDIED ENTITY	DEFINITION	RATE
Bilreiro et al. [21] (2017)	10–36 (range)	Clinical success	Complete absence of symptoms	4/6 (66.67%) for glue
				30/34 (88.24%) for coils
		Recurrence	Dilated pampiniform plexus veins (calibre >3 mm) in a supine position with reflex (with/without Valsalva manoeuvre)	3/26 (11.54%) for glue
				6/103 (5.83%) for coils
Favard et al. [8] (2015)	24.4 ± 17 (mean ± SD)	Clinical success	Absence of reflux on Doppler ultrasound examination and/or absence of scrotal pain and heaviness depending on the initial indication	83.9%
		Recurrence	Varicocele which required a new endovascular or surgical repair	7/63 (11%) for glue
				7/53 (13.2%) for mechanical agents
				4/66 (6%) for sclerosing agent
Puche‑Sanz et al. [6] (2014)	39 (median)	Clinical success	Patients who completely improved or partially improved but appeared significantly satisfied	86.9% for coils
		Recurrence	Persistence of venous reflux on ultrasonography	13.1% for coils
Sheehan et al. [5] (2020)	58 (median) (range, 28–106)	Recurrence	Recurrence of testicular pain in the presence of a varicocele	7/71 (16%) for glue and coils

The fact that only 17.24% (5 out of 29) of the patients who were initially referred for scrotal pain and who had ultrasonographic recurrence experienced pain during follow‑up suggests that ultrasonographic persistence or relapse of venous reflux does not automatically imply persistence or recurrence of scrotal pain [6]. Consequently, it is pertinent to systematically assess both clinical and ultrasonographic successes. Puche‑Sanz et al. recorded an ultrasonographic success rate (that they defined as disappearance of SVR) of 68.6% at 3–6 months [6]. In our study, we adopted the same definition and had a 44.87% rate of ultrasonographic success. D’Andrea et al. reported a similar ultrasonographic success (that they defined as absence of SVR or SVR <3 cm/s) of 69.23% at 6 months [22]. Another interesting finding of this study is that SVR was found to be the best predictor of pregnancies in subfertile couples [22]. This finding highlights the importance of evaluating and quantifying SVR during follow‑up.

Regarding semen analyses, multiple studies have shown semen quality improvement following embolization in groups of subfertile men [17–20]. Nonetheless, the benefit of varicocele repair on fertility outcomes remains inconclusive [20]. A meta‑analysis on the outcomes of varicocele repair in men from couples with otherwise unexplained fertility claimed that varicocele repair could improve a couple’s chances of obtaining a pregnancy [23]. In a recent meta‑analysis assembling 36 studies and 4,473 patients, Çayan et al. reported that natural pregnancy rates were the highest for patients who had undergone microsurgical varicocelectomy (41.97%), followed by the macroscopic inguinal varicocelectomy group (36%) and then by the varicocele embolization group (33.20%) [25]. For patients referred for infertility, paternity rates vary between 21.2% and 46.3% [18, 19, 20, 25, 26]. D’Andrea et al. reported that, in 52 men who were referred for infertility and in whom SVR disappeared on follow‑up, the spontaneous pregnancy rate reached 48.1% [22]. Disappearance of SVR on ultrasound follow‑up studies might therefore be a promising objective to achieve [22]. Anyhow, varicocele embolization can be used to improve semen quality, avoiding the need for complex assisted reproductive techniques and their potential complications [27, 28].

Evidence from other studies shows that varicocele repair is effective for restoring normal testicular growth in paediatric patients presenting for asymmetric testicular growth [9].

Complications occurred in 19/182 interventions (10.45%), all of which were self‑limited. According to the Cardiovascular and Interventional Radiological Society of Europe (CIRSE) classification, 18 were grade 1, and 1 was grade 2 (femoral artery puncture) [30].

Since varicocele embolization is usually performed in young and healthy males, it is important to minimize radiation doses [30]. Our dosimetric results were similar to those of other studies. Favard et al. reported a fluoroscopy time of 21.38 ± 9.08 min, a KERMA value of 504.60 ± 481.31 mGy and a DAP of 139.2 ± 129.2 Gy∙cm² with mechanical agents (values expressed as mean ± SD) [8]. Chalmers et al. evaluated DAP, average tube potential and screening times in a study containing a retrospective group and another prospective group [30]. By primary beam collimation to the smallest possible area, minimizing tube current, not taking spot films or angiographic runs and minimizing fluoroscopy time, a 300‑fold reduction in testicular doses and a 7‑fold reduction in DAP values were obtained [30]. These numbers should prompt interventional radiologists to adopt radiation‑reduction measures.

### Limitations

Our study presents several limitations. First of all, it is a retrospective single‑centre study. The lack of a control group or comparison groups is an additional limitation. Besides, coil embolization could hardly be compared with embolization with other embolic agents due to the low number of patients embolized with either glue or glue and coils.

Another limitation is that ultrasonographic success was evaluated as a binary variable in our study. Evaluation with quantification of SVR is preferable, as it might in fact elucidate an association between degree of reflux and clinical success rates [6].

Many parameters – such as varicocele clinical or radiological grades, ISV anatomic variations or even the impact of varicocele‑associated pain on quality of life – were not examined, largely owing to the retrospective setting of the study.

## V. Conclusions

We presented daily practice outcomes for a large series of patients at our tertiary centre, reporting on radiological success rates, clinical success rates and complications rates. In addition, we performed a dosimetric study.

Varicocele embolization remains a reliable and safe treatment option for varicocele. It seemed to be an efficient procedure for patients referred with scrotal pain.

Despite all the knowledge on varicocele and its vast prevalence in male individuals, randomized controlled trials are still warranted to elaborate individualized treatment algorithms on patient selection, optimal timing of the treatment and how the patients should be treated. Clear and strict definitions are necessary for clinical and ultrasonographic successes to better compare different techniques of varicocele repair.
